# Approach to Solid Liver Masses in the Cirrhotic Patient

**DOI:** 10.4021/gr2009.10.1314

**Published:** 2009-09-20

**Authors:** Nimer Assy, Najib Assy, Nir Samuel, Aracdi Lerman, William Nseir

**Affiliations:** a Liver Unit, Ziv Medical Center, Safed, Israel; b Technion Institute, Haifa, Israel; c Department of Radiology, Ziv Medical Centre, Safed, Israel; d Holy Family Hospital, Nazareth, Israel

**Keywords:** Liver mass, Hepatic nodule, Tumor, Lesion, Cirrhosis, Hepatocellular carcinoma, Cholangicarcinoma, Dysplasia, Metastasis, Fine needle aspiration, Core biopsy

## Abstract

Liver masses in cirrhosis are increasingly being recognized with the use of new imaging modalities. The majority of these lesions are detected by ultrasound, enhanced CT and MRI. The most likely diagnosis of a solid liver lesion in a cirrhotic liver is hepatocellular carcinoma, followed by high grade or low grade dysplastic nodule, and cholangiocarcinoma. Lymphoma and liver metastasis are extremely rare. Diagnosis is made by contrast enhanced ultrasound, multi detector (MDCT) and MRI. Fine needle core biopsy (FNCB) or aspiration (FNAB) or both may be required in doubtful cases. If uncertainty persists on the nature of the lesion, surgical liver resection is recommended. This review discusses the main characteristics of the most common solid liver masses in cirrhotic patient.

## Introduction

Liver masses are increasingly being recognized with the widespread use of imaging modalities such as ultrasonography, computed tomography, and magnetic resonance imaging [1]. The most important initial question is whether cirrhosis or an extra hepatic disorder may lead to the development of a focal liver lesion, such as a regenerative nodule, hepatocellular carcinoma, and a metastatic mass or infectious process. An accurate history and physical examination are essential to diagnosis and treatment for solid liver mass. A history of post viral cirrhosis due to HBV or HCV may point to hepatocellular carcinoma (HCC) [1]. Use of oral contraceptives or anabolic steroids can be related to hepatic adenoma [2]. Alcohol use and vinyl chloride exposure are related to angiosarcoma [3]. Primary sclerosing cholangitis, Caroli’s disease and choledochal cysts are associated with cholangiocarcinoma [4]. A previous neoplasm or chemotherapy increases the suspicion of metastatic disease. Physical examination should look for liver tenderness, lymphadenopathy, hepatomegaly, splenomegaly, ascites, other stigmata of chronic liver disease, or general deterioration signs (fever, weight loss). High alkaline phosphatases, high lactate dehydrogenase (LDH), low albumin, high prothrombine time, and iron overload are non specific but may suggest underlying cirrhosis or an infiltrative process [5].

A liver mass in a cirrhotic liver should be viewed as an HCC until proven otherwise. The diagnosis of liver masses in a cirrhotic liver includes malignant and benign lesions [6-8] (Table 1). After detecting hepatic mass on ultrasound, the mass can be characterized with contrast enhanced ultrasound, multi detector computed tomography or magnetic resonance imaging. Each technique has its advantages and disadvantages. The choice of modality depends on many factors including cirrhosis, fatty infiltration, and number of nodules. This review discusses the various characteristics of the most common solid liver masses (lesions) in cirrhotic patient including value of tumor markers, imaging techniques, fine needle aspiration or biopsy, dilemmas and pitfalls, clinical differential diagnosis of malignant and benign lesions, liver biopsy and liver mass resection.

**Table 1:** Accuracy and key features of imaging techniques in the diagnosis of most common liver masses in cirrhosis

Lesions	US- US Doppler, Contrast ultrasound	Triphasic Dynamic CT	MRI	PET SCAN	CT-Angiography
**HCC**	+ Hypo or hyper echoic Doppler: hyper vascular. index and flow high, spectral broadening	+++ hyper vascular, often irregular borders Heterogeneous> Homogeneous abnormal internal vessel Hallmark feature is arterial hypervascular- and venous wash-out SS 52-54%	+++ hyper vascular Poor different: Hypo intense T-1, Hyper intense T2; Well different: Hype intense T-1, Iso intense T-2 SS 53-78%	+ Increased uptake. but many HCC do not show uptake at PET	++++ Hyper vascular Av shunting angiogenesis
**Cholangio -CA**	Bile duct dilatation if major ducts are involved Intrahepatic CCC: no bile dilatation	Hypo dense lesion Delayed enhancement	Hypo intense T1 Hyper intense T2 MRCP is useful	SS 93% Increase uptake	Hyper vascular
**Metastasis**	+ SS 40-70 % hypo echoic to hyper echoic; Doppler; low index and flow; presence of spectral broadening	+++ SS 49-74 % complete ring enhancement	+++ SS 68 -90 % Low intensity T-1 High intensity T-2	+++++ SS 90-100% colon, pancreas	++++ SS 88-95% hyper vascular
**Haemangioma**	++ Hyper echoic Doppler: low flow, low index, absence of spectral broadening	+++ Peripheral puddles, fill in from periphery, enhancement on delayed scan	++++ Peripheral enhancement centripetal progression HyperintenseT2, hypo intense T1 SS >95%, SP 95%	No uptake	+++ Cotton wool pooling of contrast, normal vessels without AV shunt, persistent enhancement
**Focal fatty liver**	+ hyper echoic, no mass effect, no vessel displacement	++ Sharp interface Low density (<40u)	+++	No uptake	normal finding
**Adenoma**	+ Heterogeneous Hyper echoic If haemorrhage: anechoic centre. Doppler: variable flow and spectral broadening	++ Homogenous>Heterogeneous, Peripheral feeders filling in from periphery	++ Capsule, Hyper intense T1 (intra lesion fat )	no uptake uptake if degeneration to HCC	++ Hyper vascular Large peripheral Vessel. Central scar if haemorrhage

+, degree of accuracy; SS sensitivity; SP specificity; a; Intraoperatrive ultrasound, contrast ultrasound and EUS are highly sensitive to detect liver mass; From Assy N, World J Gastroenterol. 2009;15:3217-27.

## Tumor markers

Alpha fetoprotein (AFP), PIVKA-II (protein induced by vitamin K absence or antagonist II), desgamma-carboxy prothrombin (DCP) are tumor markers for HCC [9]. The combination of Ca 19-9 + CEA markers gave an accuracy of 86% in diagnosis of cholangiocarcinoma [10]. AFP values higher than 400 ng/ml in the presence of cirrhosis is indicative of HCC. Thirty percent of patients with HCC less than 2 cm have normal AFP, twenty percent of HCC do not produce AFP, and levels between 20 - 250 ng/ml are frequently seen in regenerating nodules or viral cirrhosis [11]. A rising AFP over time is virtually diagnostic of HCC. The serum level of at least 1 of the tumor markers was elevated in 88% of patients with proven malignancy, and in 57% of cases the elevation was marked. Early lesions may have elevated tumor markers in fewer than 30% of cases [12]. The sensitivity, specificity, positive and negative predictive value of tumor markers in the diagnosis of HCC is shown in Table 2 [9].

**Table 2:** Accuracy of tumor markers in the diagnosis of HCC

	Normal value	Sensitivity %	Specificity %	PPV %	NPV %	Diagnostic accuracy %
**AFP (ng/dL)**	20	55	97	95	69	77
**CEA (ng/dL)**	7	22	78	48	51	51
**TPA (U/L)**	90	70	61	62	70	66
**DCP (AU/ml)**	0.09	53	88	88	66	71

Des-gamma-carboxy prothrombin (DCP). Tissue polypeptide antigen (TPA), alpha fetoprotein (AFP), carcinoembryonic antigen (CEA), From Grazi GL, liver transplantation and surgery 1995;1: 249-255.

The limitation of developing HCC markers is probably due to heterogeneity assay methods and result reporting, limited analysis of demographics and causes of liver disease as covariates in the expression of these biomarkers. In addition, these molecules need to be validated and cost-effectiveness especially those markers proposed as diagnostic or prognostic role. Further studies are needed to confirm the roles and to validate these biomarkers.

## Imaging techniques

Key features of imaging technique in the diagnosis of liver mass are shown in Table 1.

The gold standard for detection and location of focal lesions in cirrhosis is enhanced MRI or triple phase dynamic spiral CT [13]. Conventionally a triple phase CT scan includes unenhanced, arterial and venous phases. The fourth phase is a delayed venous scan (quadruple phase multi detector computed tomography, MDCT) [13]. This is only required for small lesions thought to be HCC or cysts and hemangiomas. A single imaging modality can be sufficient in cases such as metastasis which show interval development or progression. CT Porto angiography is one of the most sensitive imaging for metastasis but it is an examination that is performed in high selected cases, in few institutions and not for all types of liver lesions [14]. FDG PET CT scan is not very useful for HCC and therefore is not the best imaging modality to distinguish benign from malignant lesions [15]. Ultrasound contrast agents and MRI using iron or gadolinium contrast better detect smaller lesions, satellite lesions or distant metastasis [16-18]. Radiographic characteristics favoring hepatocellular carcinoma include the presence of a lesion with different densities, arterial hyper vascularisation and venous wash-out. The diagnostic accuracy of MRI and computed tomography (CT), sensitivity, and specificity in the diagnosis of liver mass is shown in Table 3 [19].

**Table 3:** Accuracy of magnetic resonance imaging (MRI) and spiral computed (CT) in the diagnosis of liver mass (nodule)

	MRI %	CT %
**Sensitivity**	85	70
**Specificity**	71	86
**Positive predictive value**	92	95
**Negative predictive value**	56	43
**Diagnostic Accuracy**	82	74

From: de Ledinghen: Eur J Gastroenterol Hepatol, 2002;14:159-165.

### Fine needle aspiration and core biopsy

Fine needle aspiration and core biopsy (FNAB) is safe, accurate and cost effective. Its specificity approaches 100% and its sensitivity is 67-100% [20]. FNAB under CT or under ultrasound (in an appropriate location) is the method of choice. FNAB is superior to fine needle core biopsy (FNCB), however, the methods are complementary, i.e., FNAB and FNCB have an accuracy of 78% separately and 88% when considered in combination [21-22]. However, many pathologists would state that core biopsies are much preferred over needle biopsies for diagnosis of hepatic mass, since well differentiated HCC cannot be separated from normal liver. Complications (mostly haemorrhage) are rare with 0.5% minor complications and 0.05% major complications [23]. Another concern is the seeding of tumor. Blind FNAB is diagnostic in more than 50% of cases which increases to 65% when performing a second pass [24]. An additional 5-10% of tumors will be recognized if cell block is obtained. Markers commonly used for immunohistochemical staining in the evaluation of hepatic tumors include polyclonal CEA, cytokeratin 8/18 pair, cytokeratin 7/20, hep par 1, Glypican-3, and AFP for hepatocellular carcinoma [25]; cytokeratin 7/19, cytokeratin 7/20, B-HCG, CEA, and mucin-1 for cholangicarcinoma [26], CD 34, CD31, and factor V111 for hemangiendothelioma; and cytokeratin 7/20 for metastatic liver disease. The sensitivity and specificity of FNA cytology, needle core biopsy and combined FNA/FNCB in the diagnosis of malignant and benign liver lesions are shown in Table 4 [20].

**Table 4:** The sensitivity (%) of FNA cytology, needle core biopsy, and combined FNA/FNCB in malignant and in benign liver lesions

Biopsy Site	FNA %	FNCB %	Combined %
Liver Metastasis	86	83	88
Hepatocellular Carcinoma	100	89	100
Benign Liver Lesions	100	89	100

FNA, fine needle aspiration. FNCB, needle core biopsy, From: Stewart CJ; J Clin Pathol. 2002; 55: 93–97.

## Clinical dilemmas and pitfalls

Screening for HCC in cirrhotic liver includes ultrasound plus AFP levels every 6 months. The AASLD guidelines recommend ultrasound only [27].

### Liver mass more than 2 cm

Lesions more than 2 cm need just one imaging technique showing typical findings (enhancement in the arterial phase and washout in the portal venous phase) or one imaging technique and AFP levels higher than 400 ng/ml in order to make a non-invasive diagnosis of HCC [11]. More than 80% of over 2 cm in a cirrhotic liver are HCC [28]. An elevated AFP confirms the diagnosis. If AFP is normal, further imaging will be diagnostic (triphasic CT, MRI) [11]. If there is still doubt, FNCB may be indicated.

### Liver mass less than 2 cm

Seventy-five percent of masses which are less than 2 cm in a cirrhotic liver are HCC [29]. Lesions smaller than 2 cm are divided into larger and smaller than 1cm. The larger nodules should be diagnosed and the small lesions should be surveyed every 3 months [27, 30]. Nodules more than 1 cm but less than 2 cm (1 - 2 cm) need diagnostic workup with two coincident or serial imaging techniques (among US, CT, and MRI with IV contrast injection) rather than just proceeding with biopsy. If two of these techniques show typical imaging criteria, then it is possible to diagnose HCC. Nodules less than 1 cm need screening follow up every 3 months. A small nodule can be pre-neoplastic or benign. However, Caturelli showed that 69% of new nodules in a cirrhotic liver are malignant [29]. Moreover, liver cell dysplasia is found in 60% of cirrhotic livers containing hepatocellular carcinoma and in only 10% of non-cirrhotic livers [31]. Because of the risk of tumor seeding, biopsy should be avoided if surgical resection is possible. Nine percent to 37% of HCC are resectable at diagnosis. Contraindications to resection are: decompensated cirrhosis, extra-hepatic metastases, involvement of hepatic nodes or inferior vena cava (IVC), or bilobar extension [32].

### Increased AFP without detectable liver mass on liver imaging

In this case repeated dynamic CT or MRI every 3 months is the rule [12, 33]. An elevated AFP does not necessarily diagnose HCC, especially in patients with HCV who commonly have modest elevation of AFP without HCC. A marked AFP is helpful but modest elevations would certainly not be an indication for OLT in the absence of a liver mass.

## Clinical differential diagnosis of the most likely lesions in liver cirrhosis

### Malignant lesions

#### Hepatocellular carcinoma

HCC is a common tumor with an incidence of 1 - 6 % among cirrhotic patients [34]. Risk factors include cirrhosis, alcohol, HBV, HCV, metabolic liver diseases, environmental carcinogens, hormonal treatments and smoking [35]. About 90% - 95% of HCC arise in cirrhotic livers. Autopsy studies indicate that 20 - 40% of patients with cirrhosis have HCC. The tumor size and severity of liver disease influences survival rate. Patients with tumor less than 5 cm have a survival of 80% at 1 year and 20% at 3 years [32]. New abdominal pain, recent hepatomegaly, hemoperitoneum, persistent fever or weight loss in a cirrhotic patient should raise suspicion for HCC. Laboratory results that characterize HCC are a sudden increase in alkaline phosphatases, an increased ratio AST/ALT, an erythrocytosis, a persistent leukocytosis, recurrent hypoglycaemia, hypercholesterolemia and hypercalcemia. The last four findings are paraneoplastic manifestations [32] together with ectopic hormonal syndrome, hypertrophic osteoarthropathy, and porphyria cutana tarda [36]. Complications of HCC include obstructive jaundice, and rupture of HCC (hemoperitonium; 60 - 90% mortality)

#### Dysplastic nodule

Dysplastic nodule often occurs within regenerative cirrhotic nodules. They can show low or high grade dysplasia. A progression from regenerative nodule with low grade dysplastia, high grade dysplasia, well differentiated and poorly differentiated HCC is possible [37]. MRI best differentiates this iso-or hypo- intense lesion from hyper intense HCC [38]. In difficult cases, histology is required after liver resection or liver transplant. If HCC cannot be confirmed, repeat investigation later.

#### Cholangiocarcinoma

Cholangiocarcinoma accounts for 20% of primary liver tumors, arises as adenocarcinoma, papillary or mucinous carcinomas [39]. Risk factors are cirrhosis, primary sclerosing cholangitis (PSC, 10%), bile duct adenoma, choledochal cysts, biliary papillomatosis, Caroli's disease and liver fluke [40]. Jaundice is the most frequent clinical presentation, and rapidly increasing bilirubin associated to weight loss predicts cholangiocarcinoma [41]. Tumor markers CEA, CA-19-9 or AFP may be elevated. CA 19-9 level higher than 100 has 89% sensitivity and 86% specificity [42]. There are three anatomic subtypes: peripheral intrahepatic 15%, perihilar central (Klatskin tumor) 60% and distal common bile duct 25%. Peripheral cholangiocarcinoma resembles HCC without cirrhosis. The central hilar and distal types are associated to sclerosing cholangitis, inflammatory bowel disease or other chronic biliary disease. US and CT show marked intrahepatic duct dilatation [43]. An abrupt change in the calibre of the bile duct suggests malignancy [44]. Digital image analysis (DIA) and fluorescent in situ hybridization (FISH) are more sensitive than routine standard brush cytology in the diagnosis of cholangiocarcinoma. ERCP, percutaneous trans hepatic cholangiography (PTC) and magnetic resonance cholangiopancreatography (MRCP) assess the resectability of the tumor. PET CT stages these tumors with a sensitivity of 93%. The suggested screening includes US, CEA and CA 19-9 every 6 months, ERCP and brush cytology if there is biliary stenosis [45].

#### Lymphomas

Liver involvement is common in Hodgkin's disease including lymphoma infiltration (diffuse small nodules or large masses), drugs, viral hepatitis, and sepsis. Cholestasis is uncommon and vanishing bile duct syndrome has been described [46]. The differential diagnosis includes reactive infiltrate and T-cell lymphomas. Primary hepatic lymphoma is rare and can present as solitary or multiple masses, as a diffuse hepatic involvement with hepatomegaly, or as hepatic failure with elevated LDH [47].

#### Liver metastasis

The liver is the most common site of metastasis from the gastrointestinal tract, pancreas, breast, and lung [48]. Only 20 % of liver metastases present as solitary lesions. The involvement of both hepatic lobes is the most common. Although, liver metastasis is a rare finding in cirrhosis, on CT-scan, colorectal metastases appear as low attenuation lesions, often with irregular margins and necrotic centres [49]. During the early vascular phase of dynamic CT, metastasis appears with increased enhancement. The sensitivity of CT (85%) can be augmented by CT arterial portography [50]. Intra operative ultrasound has excellent sensitivity and specificity for colorectal adenocarcinoma metastasis [51]. The most promising imaging modality is PET CT with FDG that accumulates in cells with hyper metabolism. Colon, lung, and breast cancer can be staged with PET CT with sensitivity 92 - 100% and specificity 85-100% [52]. In metastatic colorectal carcinoma, the prognosis has improved following surgical resection. Contraindications to resection include: more than 4 liver metastases, extra hepatic spread and involvement of hepatic lymph nodes or vascular invasion. Calcified metastases from stomach, pancreas, lung and breast to the liver are extremely rare. Guided FNA will help identifying the primary lesion [53].

Other very rare malignant tumors in cirrhotic liver includes: epithelioid hemangioendothelioma, cystadenocarcinoma, and angiosarcoma, undifferentiated sarcoma of the liver, rhabdomyosarcoma, fibro sarcoma, and leiomyosarcoma.

### Benign lesions

#### Haemangioma

Haemangioma is found in 20% of the general population, more commonly in women [54]. The majority are asymptomatic. Giant haemangioma (more than 4 cm) are symptomatic in 40% of cases. Symptoms are rare and may include abdominal pain, early satiety, anorexia, nausea [55]. Contrast enhanced CT or MRI are the best modalities for the diagnosis. The risk of rupture is minimal and does not justify resection. Other complications include thrombosis, sclerosis, and calcification. The Kasabach-Meritt syndrome (consumption coagulopathy) and the Bornman-Terblanche-Blumgart syndrome (fever and abdominal pain) constitute an extremely rare complication [56].

#### Hepatic Adenoma

Adenoma occurs in women with oral contraception use more than 5 years or in diabetic patients [57]. Multiple adenomas are associated with glycogen storage disease type I and type III and adenomatosis (more than 10 adenomas) is observed with anabolic or androgenic steroids consumption [58, 59], abdominal discomfort is common. The lesion is hypo- to hyper- echoic on US and hypo- to hyper- dense on CT. MRI is not specific [60]. The lesions are often smaller than 8 cm but may be larger than 15 cm. Five percent of hepatic adenomas transform to HCC [61]. Beta-catenin immuno staining may be useful for diagnosis [62]. Spontaneous rupture and hemoperitoneum occur in 10% of cases, especially during menstruation, pregnancy or post partum. Most herpetologists advocate resection and discontinuation of oral contraception [63].

#### Focal fatty infiltration of the liver

In 10% of patients with fatty liver, fat accumulates focally or shows focal sparing, usually in the anteromedial segment of the left lobe. These patients usually have diabetes, hyperlipidemia, and obesity, drink alcohol or take steroids [64]. On US, fat is hyper echoic. On CT, it has low attenuation. Focal fatty liver does not displace intrahepatic vessels. The gold standard imaging technique is MRI with increased signal on T1 sequence [65]. Fat suppression techniques are also very promising.

Other very rare benign tumors in cirrhosis includes: hepatobiliary cyst adenoma, bile duct adenoma (cholangioma), biliary papillomatosis, lipomas, myolipomas, angiomyolipomas, schwannomas, neurofibromas and chondromas, inflammatory pseudotumor, and pseudo-lesions.

## Liver biopsy versus liver mass resection

Before hepatic resection, lesions should be measured, counted and localized to the Couinaud segments. Their relationship to major anatomic structures (portal vein, hepatic artery, inferior vena cava, hepatic vein) should be detailed [66]. If malignancy is obvious, biopsy should be avoided because of possible tumor seeding [67]. Liver histology by true cut needle biopsy is much more profitable than fine needle aspiration and cytological examination but has several disadvantages: if the tumor is small (less than 2 cm), a second attempt should be made in 20% of cases [68], bleeding is mild in 1% and severe in 0.1%. In 10 % of cases a firm diagnosis is not established and resection should be performed.

The Child-Pugh score helps selecting which patients should undergo hepatic resection. Survival depends on the regenerative potential and the presence of cirrhosis [69]. Traditionally, cirrhosis is a contraindication to hepatic resection because of high mortality rate (20%). The dilemma arises when patients with cirrhosis require a hepatic resection. The problem is that 10 - 20% of patients with cirrhosis have primary hepatic malignancy. Moreover, 80-90% of patients with HCC and 10-20% of patients with cholangiocarcinoma have cirrhosis. The operative mortality of extensive hepatic resection in patients without cirrhosis is between 1 - 10% [70].

Patients with compensated cirrhosis may benefit from liver resection, radiofrequency ablation (RFA), or transarterial chemoembolisation (TACE). Patients with decompensated cirrhosis would probably have no survival benefit [71]. In selected patients, liver transplantation may be more beneficial.

## Conclusions

There are no clear cut indications when to use which imaging modality in differentiating benign from malignant lesion. Choice of modality depends on many factors including cirrhosis, steatosis, and the number of nodules. The most likely diagnosis of liver mass in cirrhotic patients is HCC, followed by high and low grade dysplastic nodule, and cholangiocarcinoma. Ultrasound is often the first diagnostic imaging and contrast enhanced US may answer your question. MRI or CT often allows the correct diagnosis of a primary liver tumor. PET/CT using FDG mainly used to exclude regional and distant metastasis in primary liver tumors. In cirrhotic liver, main objective of the biopsy of a nodule less than 2 cm is to ascertain a diagnosis of HCC, which is now improved by the use of specific molecular markers. In non-cirrhotic liver, liver biopsy is required for the differential diagnosis of liver cell adenoma with HCC, and cholangiocarcinoma with liver metastasis, respectively.

## Abbreviations

FNAB: Fine needle aspiration biopsy
FNCB: fine needle core biopsy, HCC, hepatocellular carcinoma
AFP: alpha-fetoprotein
CT: computed tomography
MRI: magnetic resonance imaging
DN: dysplastic nodule
RN: regenerative nodule
FNH: focal nodular hyperplasia
FFL: focal fatty liver
DN: dysplastic nodule
RN: regenerative nodule
CP: Child-Pugh's score, OLT, orthotropic liver transplantation
AFP: alpha feto protein
NRH: Nodular Regenerative Hyperplasia
PNT: Partial Nodular Transformation
RFA: radiofrequency ablation
PEI: percutaneous ethanol injection
(TACE): hepatic arterial chemoembolisation
DIA: Digital image analysis
FISH: fluorescent in situ hybridization
